# Establishment and Characterization of Patient-Derived Xenografts (PDXs) of Different Histology from Malignant Pleural Mesothelioma Patients

**DOI:** 10.3390/cancers12123846

**Published:** 2020-12-20

**Authors:** Roberta Affatato, Paolo Mendogni, Alessandro Del Gobbo, Stefano Ferrero, Francesca Ricci, Massimo Broggini, Lorenzo Rosso

**Affiliations:** 1 Laboratory of Molecular Pharmacology, Istituto di Ricerche Farmacologiche Mario Negri IRCCS, 20156 Milan, Italy; roberta.affatato@marionegri.it (R.A.); francesca.ricci@marionegri.it (F.R.); 2 Thoracic Surgery and Lung Transplant Unit, Fondazione IRCCS Ca’ Granda-Ospedale Maggiore Policlinico, 20122 Milan, Italy; paolo.mendogni@policlinico.mi.it; 3 Division of Pathology, Fondazione IRCCS Ca’ Granda-Ospedale Maggiore Policlinico, 20122 Milan, Italy; alessandro.delgobbo@policlinico.mi.it (A.D.G.); stefano.ferrero@unimi.it (S.F.); 4 Department of Biomedical Surgical and Dental Sciences, University of Milan, 20122 Milan, Italy

**Keywords:** malignant pleural mesothelioma, in vivo models, patient-derived xenografts, cell lines, 3D cultures

## Abstract

**Simple Summary:**

Malignant pleural mesothelioma (MPM) is a rare tumor with unfavorable prognosis for which new therapeutic interventions are urgently needed. The aim of our study was to develop a preclinical model representative of the different histotypes found in human tumors that can be used as models for the discovery of new treatments and combinations. We successfully generated patient-derived xenografts (PDXs) from MPM, which strongly resembled the tumors of origin in terms of morphology and immunohistochemistry. These tumors, when growing in mice, poorly respond to cisplatin, a finding that aligned with the clinical results. From one of the PDXs, we generated 2D and 3D cultures maintaining the phenotypical characteristics of human tumors and PDXs. Altogether, these preclinical models represent a useful tool for the discovery of new targets and drug combinations.

**Abstract:**

Background: Malignant pleural mesothelioma (MPM) is a very aggressive tumor originating from mesothelial cells. Although several etiological factors were reported to contribute to MPM onset, environmental exposure to asbestos is certainly a major risk factor. The latency between asbestos (or asbestos-like fibers) exposure and MPM onset is very long. MPM continues to be a tumor with poor prognosis despite the introduction of new therapies including immunotherapy. One of the major problems is the low number of preclinical models able to recapitulate the features of human tumors. This impacts the possible discovery of new treatments and combinations. Methods: In this work, we aimed to generate patient-derived xenografts (PDXs) from MPM patients covering the three major histotypes (epithelioid, sarcomatoid, and mixed) occurring in the clinic. To do this, we obtained fresh tumors from biopsies or pleurectomies, and samples were subcutaneously implanted in immunodeficient mice within 24 h. Results: We successfully isolated different PDXs and particularly concentrated our efforts on three covering the three histotypes. The tumors that grew in mice compared well histologically with the tumors of origin, and showed stable growth in mice and a low response to cisplatin, as was observed in the clinic. Conclusions: These models are helpful in testing new drugs and combinations that, if successful, could rapidly translate to the clinical setting.

## 1. Introduction

Malignant mesothelioma is a rare but extremely aggressive and deadly cancer originating from the mesothelial cells that line the pleural or peritoneal cavities [1]. The most common form of mesothelioma is malignant pleural mesothelioma (MPM), which is mainly related to asbestos exposure [2]. Simian virus 40 (SV40) could also represent a cofactor in the development of mesothelioma, although its role remains controversial [3]. MPM has represented a clinical challenge due to limited therapeutic options, resistance to therapy, the complexity of the tumor anatomy, and late diagnosis. Median overall survival is about a year from diagnosis [2,4]. Currently, first-line standard treatment includes chemotherapy based on a doublet of pemetrexed and cisplatin or carboplatin. Recently, the vascular endothelial growth factor (VEGF) antibody (bevacizumab) was added to this standard of care on the basis of a Phase 3 trial (mesothelioma avastin cisplatin pemetrexed study (MAPS)), which showed that this combination improved the overall survival of patients [5]. Several studies attempted to define a role for immune checkpoint inhibitors in MPM [6]. Nivolumab, an antibody targeting Programmed cell death-1 (PD-1), was recently approved as a second-line treatment in Japan on the basis of the results of a Phase 2 trial [7]. MPM is a heterogeneous disease on the molecular, histological, and clinical levels, with high variability among patients [6]. Histologically, pathologists have classified MPM into three major types: epithelioid (the most frequent), sarcomatoid, and biphasic (a combination of epithelioid and sarcomatoid) [8]. The epithelioid histotype is characterized by the proliferation of atypical and malignant mesothelial cells, with a round nucleus, eosinophilic nucleoli, and a variable amount of cytoplasm. Cells show an infiltrative pattern through the mesothelial layers and the thoracic adipose tissue. The sarcomatoid histotype shows spindle cells with atypical and pleomorphic nuclei with the same infiltrative pattern. The biphasic type, as said before, is represented by a mixture of both epithelioid and sarcomatoid cells. Tumoral cells are usually reactive for immunohistochemical antibodies anti-calretinin, Wilms tumor 1 (WT-1), and cytocheratins; sarcomatoid phenotype can be highlighted with anti-vimentin staining. Recent works demonstrated how BRCA1-associated protein-1 (BAP1) is expressed in nearly one-third of malignant mesotheliomas [9]. In addition, p53 expression was evaluated on both tissue and cytological samples, but did not appear to reach sufficient diagnostic adequacy in the distinction between malignant and benign mesothelial lesions [10]. Different histological subtypes are associated with different survival in patients: better survival (12–27 months) in patients with an epithelioid mesothelioma, poor prognosis in patients with a sarcomatoid tumor (7–18 months), and intermediate survival (8–21 months) in patients with a biphasic mesothelioma [1]. On the molecular level, MPM is characterized by genetic, chromosomic, and epigenetic alterations [6,11]. The main chromosomal rearrangements and mutational events involve tumor suppressors, inactivation, and oncogene amplification, mediated by single-nucleotide variants, gene fusions, copy number losses, and splicing alterations. Among commonly mutated tumor-suppressor genes are CDKN2A, BAP1, and NF2, and, less frequently, TP53, SETD2, and LATS2 [12,13]. Considering the rarity of this tumor and the limited therapeutic strategies currently available, much effort is needed to evaluate and develop new therapeutic approaches. Preclinical models such as patient-derived xenografts (PDXs), primary tumor-derived cell lines, and 3D cultures (spheroids) of MPM are particularly useful to better understand the biology of this cancer and to identify new therapies. To date, few studies reported the relevance of PDXs as a preclinical model of malignant mesothelioma [4,14]. In this study, we established different PDX models from tumor samples obtained from patients with MPM. Here, we report the histological, biological, and pharmacological characterization of three PDXs belonging to the three different histotypes found in patients.

## 2. Results

### 2.1. PDX Development from MPM Patients

We collected 29 tumor samples from patients with MPM, which were transplanted in immunodeficient mice. The distribution of the histotypes was as expected, with the epithelioid histotype as the most frequent. The onset of tumors at first passage was variable, and some tumors took more than 9 months to appear. On this basis, we considered a lag time of 1 year from implantation to define a tumor as not growing. To further define a PDX as established, we considered at least three successful consecutive passages in the animals. With those fixed parameters, 5 implants are still under evaluation for their growth (first passage), and 2 models out of 24 were established (engraftment rate of 17%) and successfully maintained through multiple transplantation. Two had sarcomatoid histology, one epithelioid, and one biphasic. We aimed to have at least one model for each histology, and decided to further characterize three PDXs, namely, MESO3, MESO4, and MESO15, representative of sarcomatoid, biphasic, and epithelioid MPM, respectively. Table 1 summarizes the characteristics of the MPM patients and relative xenograft tumors stabilized.

### 2.2. Biological, Histological, and Immunohistochemical Analysis

**Figure 1:**
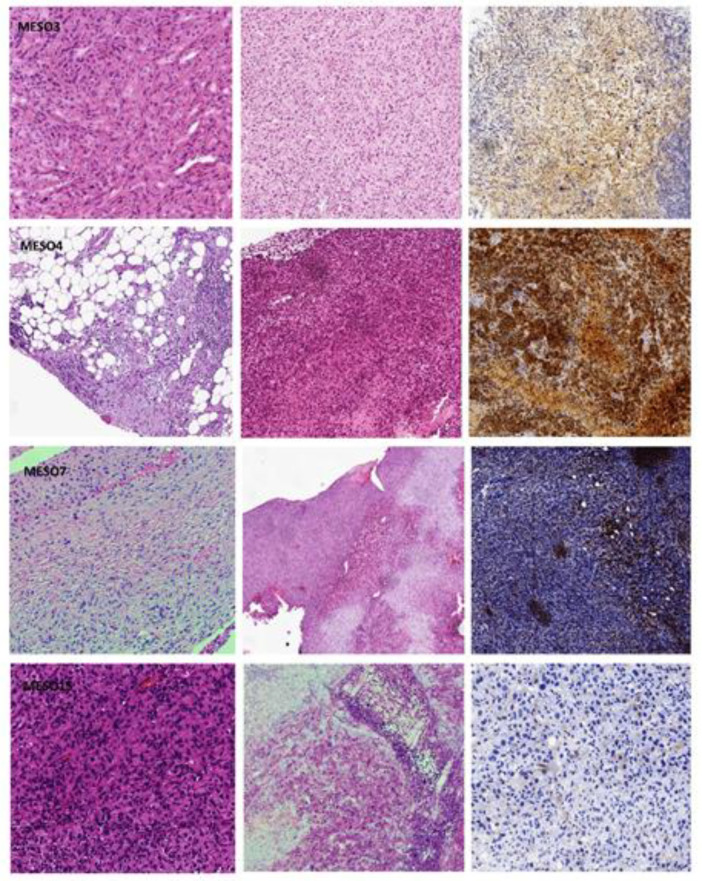
Histological and immunophenotypical features of MESO3, MESO4, MESO7, and MESO 15. Images are taken at 10× magnification. In each row, the first hematoxylin and eosin is referred to as the original tumor, the second to the PDX tumor, and the immunohistochemical stain with calretinin confirmed the mesothelial lineage on the PDX (original magnification: 100×).

All cases under study preserved good morphology, and histological features overlapped with the original tumor, showing epithelioid or sarcomatoid differentiation. Notably, in all histotypes, the immunophenotype was preserved, and vitality, as highlighted by the Ki67 proliferation index, was nearly the same as that of the original tumor (in the range of 40–70%; Table 1 and Figure 1).

In each row, the first hematoxylin and eosin is referred to as the original tumor, the second to the PDX tumor, and the immunohistochemical stain with calretinin confirmed the mesothelial lineage on the PDX (original magnification: 100×).

The established PDXs were also compared to the original tumors in terms of BAP1 and p53 expression (Figure S1). Good correlation in terms of expression was found for all samples except for MESO 15 for BAP1, which was strongly positive in the original tumor, and slightly positive in the PDX.

Different PDXs were evaluated for their growth at different passages. We calculated the slope from the growth curves, and from this the doubling time at different passages. As shown in Table 2, from the first passage, there was an increase in tumor growth rate in two models (epithelioid and biphasic), while the sarcomatoid one had fast growth from the first passage. From the second passage, growth rate remained almost unchanged in the subsequent passages for all the PDXs.

### 2.3. MPM PDX Response to Cisplatin Treatment

**Figure 2:**
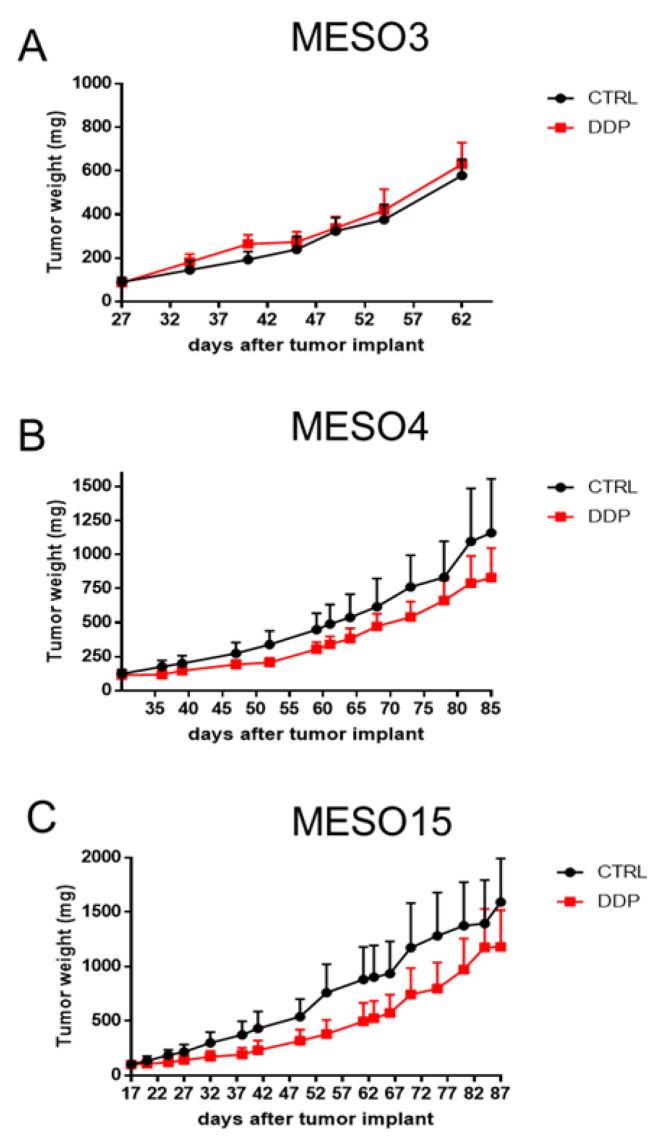
Cisplatin antitumor activity in MPM xenograft models. Tumor-bearing nude mice MESO3 (**A**), MESO4 (**B**), and MESO15 (**C**) were treated (red line) or not (black line) with DDP (5 mg/kg, iv q7dx3). Graphs represent mean ± SE of each group (5 mice per group).

Three of the four established models, belonging to the three different histotypes found in patients, were pharmacologically characterized for their response to cisplatin (DDP). As shown in Figure 2, none of the PDXs showed tumor regression after DDP treatment. MESO3 (sarcomatoid) did not show any response—there was even a faster progression after treatment—while moderate growth reduction was observed for MESO4 (biphasic), and slightly more for epithelioid MESO15.

However, as reported in Table 3, none of the PDXs showed a T/C lower than 42, which was considered the threshold to have a significant response to treatment.

The obtained results are in line with clinical data retrievable from the three patients. In fact, the patient from whom we derived MESO3, which was progressing under treatment with DDP, died three months after surgery. The patient from whom MESO15 was derived, with the PDX slightly responding to DDP, was the one with the longer survival, although it was in progression under first-line therapy.

### 2.4. MPM In Vitro Studies

**Figure 3:**
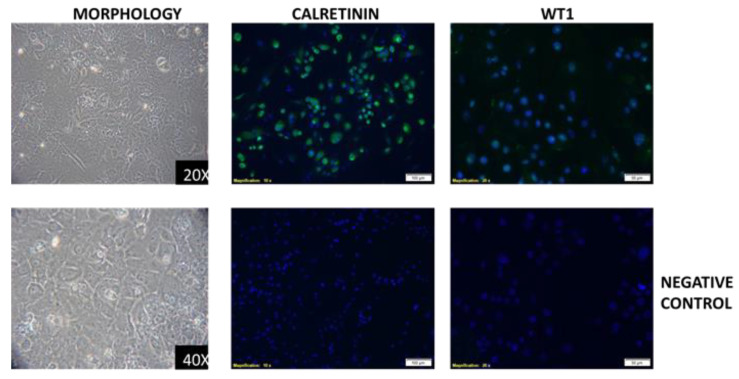
Morphology of MESO15 cell line (left), and positivity for calretinin and WT1 markers (central and left panels, respectively).

We were only able to obtain an immortalized cell line from the epithelioid MESO15 PDX. The cell line with a typical epithelioid morphology (Figure 3) was checked for the expression of the markers detected in both the tumor of origin and in the PDX, and was found to express the same markers, calretinin and WT1 (Figure 3).

**Figure 4:**
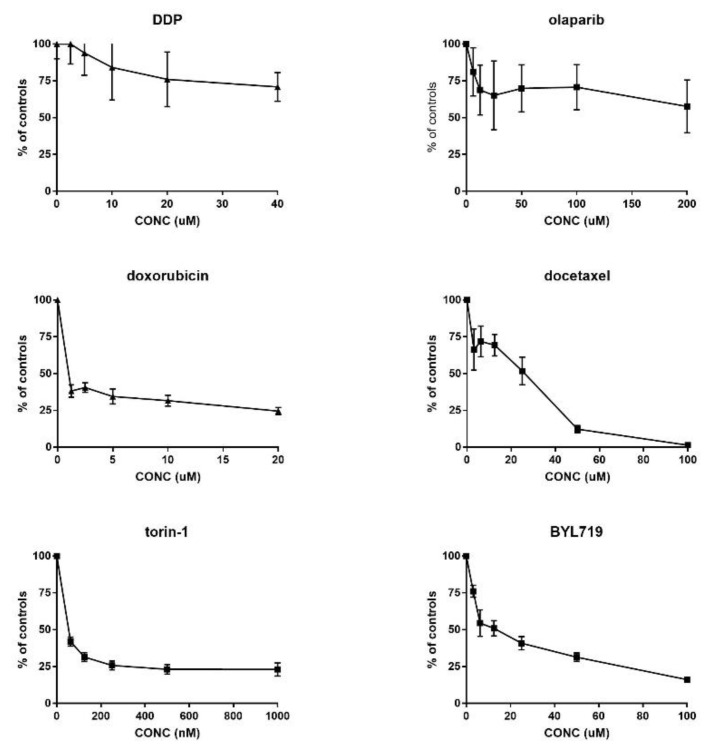
In vitro growth inhibition induced by different drugs acting with different mechanisms of action in MESO15 cell line. Data reported as percentage of controls, and values are mean and SD of six replicates per point.

This in vitro stabilized cell line was used to determine, in vitro, the activity of compounds acting with different mechanisms of action (results reported in Figure 4).

Cells did not respond well to cisplatin (in agreement with in vivo data) and olaparib, for both of which we could not determine a concentration that was able to reduce growth by 50%. Interestingly, we observed a good response to clinically used drugs such as doxorubicin, docetaxel, and to new drugs acting on the PI3K/akt/mTOR pathways, such as BYL-719 (an inhibitor of the alpha isoform of PI3K) and torin-1 (an inhibitor of mTOR).

**Figure 5:**
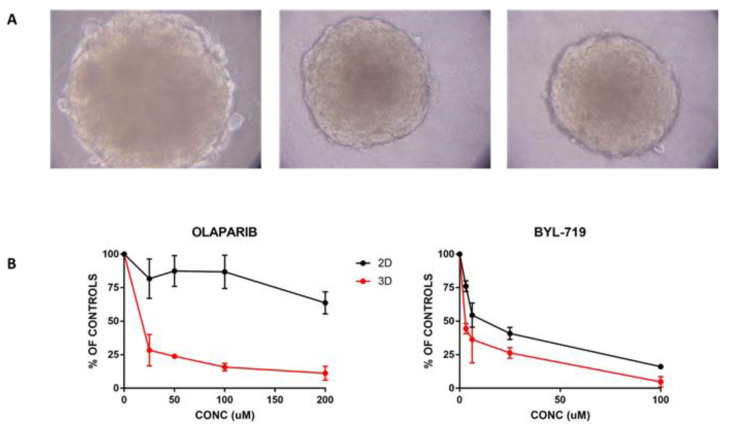
In vitro 3D growth and response to treatment. (**A**) Representative images (taken at 40× magnification) of 3D spheres obtained by plating MESO15 cells in ULA plates. (**B**) Comparison of response of MESO15 cells to BYL719 and olaparib in cells growing as monolayer (black line) or 3D spheres (red line). Data are mean and SD of six replicates.

We successfully grew this cell line in 3D, and the typical aspect of the structure is reported in Figure 5A.

Using these 3D growing cells, we compared activity in 2D vs. 3D, selecting one drug that showed activity in 2D (BYL719) and one with no activity (olaparib). Figure 5B shows that BYL also confirmed its activity in 3D, where it was slightly more active than it was in the same cells growing in monolayer. In contrast, olaparib showed a good concentration response curve in 3D, while in 2D, the drug did not show appreciable activity. The estimated concentrations that were able to reduce the growth by 50% (IC50) for BYL719 were 15 and 7 micromolar for cells growing in 2D and 3D, respectively. For olaparib, the difference was striking, with an IC50 200 μM when cells were in monolayer, and roughly 13 μM for the same cells growing in 3D.

## 3. Discussion

We reported the isolation and characterization of three PDXs from MPM covering the three different histotypes found on the clinical level for this tumor. The generation and characterization of preclinical models of cancer, recapitulating as much as possible the tumors of origin, are an important tool for the discovery of new therapeutic intervention to be translated into the clinic. This is particularly important for rare tumors that typically lack robust models. MPMs are relatively rare, aggressive tumors for which new therapeutic strategies are particularly warranted due to the very poor prognosis these patients have [15,16,17,18,19,20]. Data obtained so far with immunotherapy (in particular with the use of immune checkpoint inhibitors) were below the expectancies [7,21,22,23,24,25], particularly when we consider that their use vastly improves the outcome in other tumor types [26,27,28,29,30]. One of the factors limiting the discovery of new drugs and/or combinations potentially active in MPM is the scarce availability of models able to recapitulate the malignancy. In fact, there were several cell lines isolated from patients with MPM whose growth in vitro and response was evaluated in different laboratories [1,31,32], and some of these were also grown as organoids in 3D [31,32,33]. We demonstrated that the in vitro response in cells growing in monolayer does not always reflect the situation in more complex systems. The strong differential activity of olaparib in 3D vs. 2D growing cells was particularly intriguing, and was not in agreement with the finding obtained with other cellular systems and other drugs [34,35,36,37,38]. Olaparib, a drug currently under investigation in different solid tumors [39,40,41,42,43], is a potential candidate for MPM due to the reported mutations in the BRCA1-associated protein (BAP-1) gene found in MPM. In fact, it could represent an additional case of synthetic lethality, as it has been widely reported for poly(ADP-ribose) polymerase (PARP) inhibitors in BRCA1-mutated tumors [44,45]. In MPM cell lines growing as monolayer, the response to olaparib did not correlate with BAP1 status [46]. On the basis of our results, more complex systems should complement cell lines growing in culture as monolayer for the better definition of drugs of potential clinical use.

In this context, the availability of the same model of MPM growing in monolayer, 3D, and in vivo is a further advantage, and the possibility to screen in these models new drugs or combinations accelerates translatability to the clinic. However, few in vivo models are available, and the characterization of new ones is warranted [4,14]. We could generate four MPM PDXs (three of which characterized), derived from the three different MPM histotypes. The rate of success was relatively modest (17%), but we considered that we used immunodeficient mice lacking T cells, yet preserving B and NK cells, in our experiment, but not more severe immunodeficient strains (such as SCID or NOD/SCID), as reported by another group [14]. This percentage, however, is in line with what we reported for ovarian cancer PDX in the same mouse strain [47,48]. The PDXs that we obtained were passed for several passages in vivo, maintaining the characteristics that they had at initial passages. These models recapitulated well the molecular characteristics of the tumors of origin and, more importantly, the poor response to chemotherapy (in this case, DDP). They maintained the same characteristics during several passages in vivo as well as the doubling time that remained stable from the second passage on. They represent, therefore, a good proxy to study new possible interventions and new targeted therapies on the basis of available evidence arising from the molecular characterization of MPM tumors [12,13,49,50,51].

## 4. Materials and Methods

### 4.1. Patients and Tissue Samples

The study design for tissue collection and clinical information was approved by the Institutional Review Board of Fondazione IRCCS Cà Granda Ospedale Maggiore Policlinico (protocol number: 563_2018, 17 July 2018) and all patients provided written informed consent allowing for the storage and use of tumor samples for research purposes. Surgical tissue samples were evaluated by a pathologist for diagnosis, including immunohistochemical staining to identify the histological subtype of mesothelioma.

### 4.2. Surgical Procedure

Surgical samples were prospectively collected during surgical procedures in consecutive patients who had undergone diagnostic or therapeutic thoracic surgery from March 2017 to May 2020.

Diagnostic thoracic surgery was performed by video-assisted thoracic surgery (VATS) or minithoracotomy, under general or local anesthesia and deep sedation depending on the general conditions of the patient.

Therapeutic thoracic surgery consisted of pleurectomy and pleural decortication almost exclusively performed by a posterolateral thoracotomy.

In every case of surgery, the possibility to collect a sample for the scientific purpose of the present study was carefully evaluated following the “leftover tissue” principle, considering the absolute priority of the diagnosis. According to this, in some cases, it was not possible to collect an adequate specimen required for research.

During the study period, a total of 46 patients were submitted to surgery for histological/cytological, proven or clinically suspected MPM. Of them, 35 samples of pleura were obtained from 35 patients. Six were excluded from analysis because of the final histology, which did not confirm MPM. The 29 remaining samples were subclassified as follows: epithelioid MPM (*n* = 17), sarcomatoid MPM (*n* = 7), biphasic MPM (*n* = 5).

### 4.3. Histopathological Analyses

Tissue specimens (original tumors and PDX tissue after animal sacrifice) were formalin-fixed and paraffin-embedded (FFPE). Consecutive 4 micron thick sections were cut from each tissue block, one was stained with hematoxylin and eosin, and the other sections were subjected to immunohistochemical staining for WT1, calretinin, Ki67, BAP1, p53, and other antibodies if required for diagnosis (e.g., vimentin to corroborate the sarcomatoid differentiation, see Table 1) using automatic system BenchMark XT (Ventana Medical Systems, Oro Valley, AZ, USA). Reactions were revealed using UltraView^TM^ Universal DAB, a biotin-free, multimer-based detection system, according to the manufacturer’s instruction. Positive and negative controls were included in each slide run.

FFPE samples were collected at each passage of the PDXs and histologically compared with the original FFPE block of the patient.

### 4.4. Establishment of PDX Models

Female NCr-nu/nu mice obtained from Envigo Laboratories were used when they were 6 to 8 weeks old. Mice were maintained under specific pathogen-free conditions, housed in isolated vented cages, and handled using aseptic procedures. The Istituto di Ricerche Farmacologiche Mario Negri IRCCS adhered to the principles set out in the following laws, regulations, and policies governing the care and use of laboratory animals: Italian Governing Law (D. lg 26/2014; authorization no.19/2008-A issued 6 March 2008 by the Ministry of Health); Mario Negri Institutional Regulations and Policies providing internal authorization for persons conducting animal experiments (Quality Management System Certificate: UNI EN ISO 9001:2008, reg. no. 6121); the National Institute of Health (NIH) Guide for the Care and Use of Laboratory Animals (2011 edition) and EU directive and guidelines (European Economic Community (EEC) Council Directive 2010/63/UE). An institutional review board and the Italian Ministry of Health approved all the in vivo experiments performed with PDXs(approval 224/2018-PR of 14 march 2018). Fresh samples were subcutaneously implanted in both flanks of nude mice within 24 h from surgical removal. Each PDX model was passaged up to 5 times, after which the model was considered stabilized. At each passage, the tumor was cryopreserved (RPMI medium 40% Fetal bovine serum (FBS) and cryoprotective medium), snap-frozen in dry ice, and fixed in neutral-buffered formalin for histologic examination.

### 4.5. In Vivo Studies

PDX tumor fragments (2 × 2 mm) were subcutaneously implanted into nude mice through trocar needles and mice were randomized when the average tumor size was about 100–120 mg (5 per group). Cisplatin (DDP, Sigma-Aldrich, Milan, Italy) was given intravenously at the dose of 5 mg/kg once a week for three times (q7d × 3). Tumor growth was measured twice a week with a Vernier caliper, and tumor weights (mg = mm^3^) were calculated as follows: (length (mm) × width (mm)^2^)/2. The efficacy of the treatment was expressed as best tumor growth inhibition (%T/C = (tumor weight mean of treated tumors/tumor weight mean of control tumors) × 100). A T/C% value 42 was indicative of drug activity.

### 4.6. Establishment of PDX Cell Line

Fragments from tumors that were growing in nude mice were minced and digested with collagenase at 37 °C. After filtration, cell suspensions were washed and seeded in 6 well plates in MEM medium supplemented with 10% FBS. Attached cells were allowed to grow and, at confluence, detached, and cancer cells separated from fibroblasts and stromal cells by negative selection using MACS columns. Purified cancer cells were seeded again, and the selection was repeated. Cells were then passed for at least 5 passages, after which they were considered immortalized. To check for the expression of markers present in the original tumor and in the PDX, cells were seeded in 24 well plates with glass coverslips. When at 70% confluence, cells were fixed in paraformaldehyde, washed in Phosphate buffered saline (PBS), and stored at 4 °C. Immunofluorescence for calretinin and WT1 was performed using procedures recommended by the manufacturer. The used antibodies were antirabbit polyclonal for calretinin (SWant 7697, Marly, Switzerland) and rabbit monoclonal for WT1 (Abcam ab89901, Cambridge, UK). The same cells were grown in 3D, seeding them in ultralow attachment plates (Corning Inc., New York, NY, USA).

For cytotoxicity experiments in vitro, cells were seeded in 96 well plates and, after 24 h, treated with increasing concentrations of drugs. After 72 h, cell viability was determined using MTS assay (Promega, Milano, Italy) as described [52]. For cells growing in 3D, Cell Titer Glo Luminescent Cell Viability Assay (Promega, Italy) was used following the manufacturer’s instructions.

The drugs used for these experiments were: cisplatin (DDP, DNA interacting agent), olaparib (PARP inhibitor), docetaxel (microtubules interfering agent), doxorubicin (DNA intercalating agent), BYL-719 (PI3K alpha inhibitor), and torin-1 (mTOR inhibitor). Stock solutions were prepared in DMSO for all the drugs. Dilutions from stock solutions were performed in culture medium. Concentration-dependent curves were plotted as percentages relative to untreated controls, at 8 replicates for each time point. The mean and SD are presented. Concentrations inhibiting the growth by 50% (IC50) were calculated from the curves using GraphPad Prism Version 7.

## 5. Conclusions

We generated and characterized three PDXs representing the different MPM histotypes. These models, recapitulating well the tumors of origin, are expected to be instrumental for the discovery of new potential treatments and combinations.

## Figures and Tables

**Table 1:** Derived characteristics of malignant pleural mesothelioma (MPM) patients and patient-derived xenografts (PDXs).

	Patient		PDX	
ID	Histology	Positive Marker	Histology	Positive Marker
**MESO 3**	sarcomatoid	Vimentin, Cytokeratins AE1 and AE3	sarcomatoid	Vimentin, Calretinin, Ki67 = 60%
**MESO 4**	biphasic	Calretinin, Vimentin and WT1 (focal)	biphasic	Vimentin, Calretinin, Ki67 = 70%
**MESO 7**	sarcomatoid	Vimentin, Cytokeratins AE1 and AE3 and WT1 (focal)	sarcomatoid	Vimentin, Calretinin, Ki67 = 60%
**MESO 15**	epithelioid	Calretinin, WT1 (focal) and Vimentin	epithelioid	Vimentin, Calretinin, Ki67 = 40%

**Table 2:** Doubling times of PDXs at different in vivo passages.

	Doubling Time (Days)		
In Vivo Passages	MESO3	MESO4	MESO15
I	16.0	43.1	23.6
II	−	12.6	10.0
III	7.7	14.1	10.4
IV	7.5	13.6	14.3
V	−	−	13.3

**Table 3:** Antitumor activity in MESO3, MESO4, and MESO15.

ID	Histotype	T/C % (Day)
MESO3	Sarcomatoid	104 (49)
MESO4	Biphasic	61 (52)
MESO15	Epithelioid	50 (54)

## Supplementary Materials

The following are available online at https://www.mdpi.com/2072-6694/12/12/3846/s1, Figure S1: Expression of p53 and BAP1 in PDX tumor and in the original patients’ tumors.
