# Supplementary material for: Establishment and Characterization of Patient-Derived Xenografts (PDXs) of Different Histology from Malignant Pleural Mesothelioma Patients
Source: Cancers (Basel). 2020 Dec 20;12(12):3846. doi: 10.3390/cancers12123846 (PMC7766019; doi:10.3390/cancers12123846)
Supplement: Supplementary file 1 [file cancers-12-03846-s001.pdf]

Supplementary Materials: Establishment and Characterization of Patient-Derived Xenografts (PDXs) of Different Histology from Malignant Pleural Mesothelioma Patients

Roberta Affatato, Paolo Mendogni, Alessandro Del Gobbo, Stefano Ferrero, Francesca Ricci, Massimo Broggini and Lorenzo Rosso

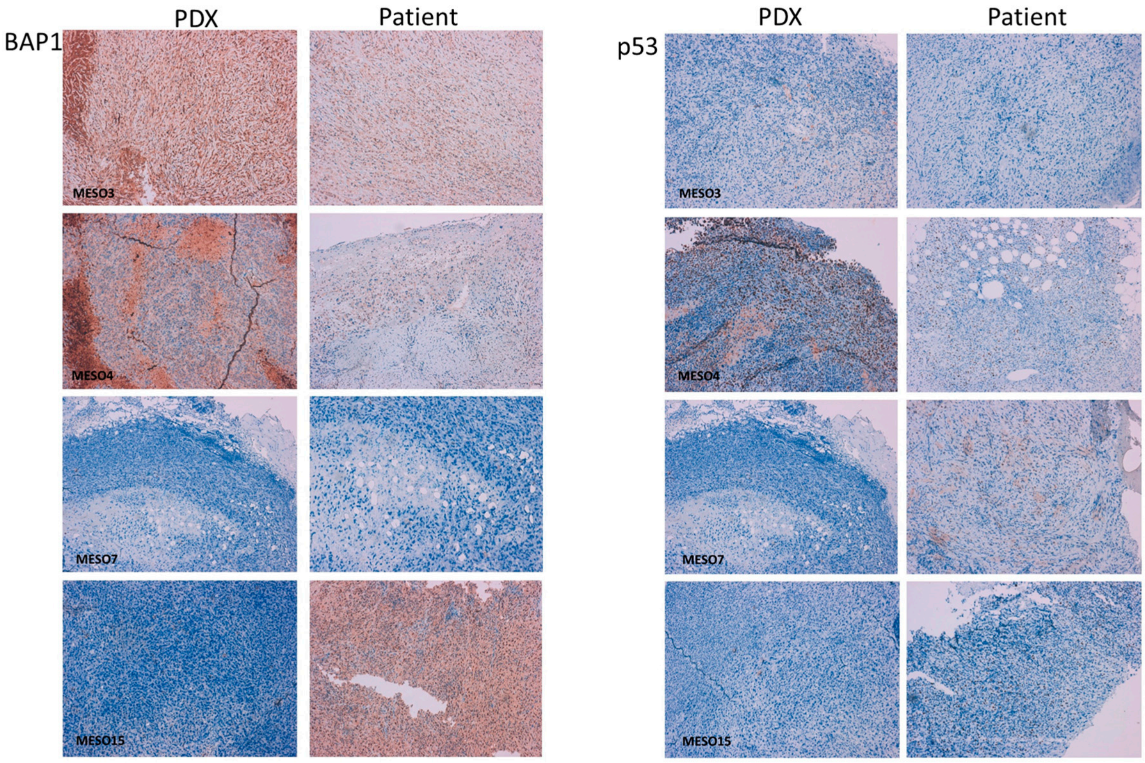

Figure 1. Expression of p53 and BAP1 in PDX tumor and in the original patients' tumors.

Publisher's Note: MDPI stays neutral with regard to jurisdictional claims in published maps and institutional affiliations.

© 2020 by the authors. Licensee MDPI, Basel, Switzerland. This article is an open access article distributed under the terms and conditions of the Creative Commons Attribution (CC BY) license (http://creativecommons.org/licenses/by/4.0/).
